# Quantifying adsorption-induced deformation of nanoporous materials on different length scales

**DOI:** 10.1107/S1600576717012274

**Published:** 2017-09-14

**Authors:** Roland Morak, Stephan Braxmeier, Lukas Ludescher, Florian Putz, Sebastian Busch, Nicola Hüsing, Gudrung Reichenauer, Oskar Paris

**Affiliations:** a Institute of Physics, Montanuniversität Leoben, Franz-Josef Strasse 18, Leoben, 8700, Austria; b Bavarian Center for Applied Energy Research, Magdalene-Schoch-Strasse 3, Würzburg, 97074, Germany; c Chemistry and Physics of Materials, Paris Lodron Universität Salzburg, Jakob-Haringer-Strasse 2a, Salzburg, 5020, Austria; d German Engineering Materials Science Centre (GEMS) at Heinz Maier-Leibnitz Zentrum (MLZ), Helmholtz-Zentrum Geesthacht GmbH, Lichtenbergstrasse 1, Garching bei München, 85747, Germany

**Keywords:** mesoporous materials, small-angle neutron scattering, dilatometry, adsorption-induced deformation, adsorption isotherms

## Abstract

A new *in situ* setup combining small-angle neutron scattering (SANS) and dilatometry was used to measure water-adsorption-induced deformation of a monolithic silica sample with hierarchical porosity. The sample exhibits a disordered framework consisting of macropores and struts containing two-dimensional hexagonally ordered cylindrical mesopores. The use of an H_2_O/D_2_O water mixture with zero scattering length density as an adsorptive allows a quantitative determination of the pore lattice strain from the shift of the corresponding diffraction peak. This radial strut deformation is compared with the simultaneously measured macroscopic length change of the sample with dilatometry, and differences between the two quantities are discussed on the basis of the deformation mechanisms effective at the different length scales. It is demonstrated that the SANS data also provide a facile way to quantitatively determine the adsorption isotherm of the material by evaluating the incoherent scattering contribution of H_2_O at large scattering vectors.

## Abstract 2

Simultaneous small-angle neutron scattering and dilatometry reveal water-adsorption-induced deformation of silica materials with hierarchical porosity along with their adsorption isotherm.

## 1. Introduction   

Nanoporous materials are used in many industrial applications exploiting solid–fluid interactions. They are used for instance in separation, catalysis, gas or energy storage, and sensing, and even for actuation (Clarkson *et al.*, 2012 ▸; Zhai *et al.*, 2011 ▸; Wang *et al.*, 2008 ▸; Davis, 2002 ▸). The latter effect is based on the mechanical deformation of the material upon the adsorption of molecules, atoms or ions (Gor *et al.*, 2017 ▸). Even though the effect has been known for almost a century (Bangham & Fakhoury, 1928 ▸), it is only in recent years that considerable progress has been made in the fundamental understanding of adsorption-induced deformation, mainly for three reasons. Firstly, model systems with tailored pore characteristics such as a monodisperse pore size and ordered as well as hierarchically organized pore arrangements can be synthesized (see Feinle *et al.*, 2016 ▸, and references therein). Secondly, new *in situ* experimental techniques have been developed to determine strains at different length scales, *i.e.* during adsorption and desorption of charges or molecules (see Gor *et al.*, 2017 ▸, and references therein). Lastly, the availability of high-quality experimental data has triggered theoretical and simulation work combining thermodynamics and elasticity theory (Gor & Neimark, 2010 ▸, 2011 ▸; Ravikovitch & Neimark, 2002 ▸).

Experimentally, there are essentially two approaches for measuring adsorption-induced deformation. Macroscopic methods such as dilatometry (Balzer *et al.*, 2011 ▸, 2015 ▸; Herman *et al.*, 2006 ▸; Reichenauer & Scherer, 2001 ▸; Amberg & McIntosh, 1952 ▸; Bangham & Fakhoury, 1928 ▸; Meehan, 1927 ▸) or ellipsometry (Boissiere *et al.*, 2005 ▸; Baklanov *et al.*, 2000 ▸) require monolithic samples or thin transparent films, respectively. A second class of methods uses X-ray diffraction of periodically ordered structures within the porous systems. Here, either the deformation of a crystalline phase forming the pore walls is measured (Shao *et al.*, 2010 ▸; Dolino *et al.*, 1996 ▸) or the deformation of an ordered arrangement of pores is detected from the corresponding shift of Bragg reflections in the small-angle X-ray scattering (SAXS) regime (Balzer *et al.*, 2015 ▸; Prass *et al.*, 2009 ▸; Günther *et al.*, 2008 ▸). Although *in situ* SAXS has proven to be a powerful technique to obtain full strain isotherms (*i.e.* the strain as a function of relative fluid pressure) of mesoporous materials with cylindrical pores on a two-dimensional hexagonal lattice, there are some fundamental restrictions. The strains are usually very small (10^−4^–10^−2^), and the analysis of Bragg peak positions may be strongly influenced by a changing scattering contrast during fluid adsorption and condensation. It has been shown by Prass *et al.* (2012 ▸) that in the capillary condensation regime a change of the form factor due to only partial pore filling can lead to apparent strains which depend on the pore size and distance distribution in a non-monotonic way. Furthermore, many ordered mesoporous materials contain some (usually disordered) micropores within the walls of the mesopores. Filling of these micropores will change the diffuse scattering part of the SAXS curve, thus potentially obscuring the exact position of superimposed Bragg peaks from the mesopores. An elegant solution to this problem is to use small-angle neutron scattering (SANS) with the effective scattering length density (SLD) of the adsorptive tuned to zero by isotope substitution (zero-SLD). The only changes in the SANS data as a function of a zero-SLD adsorbing fluid should then be due to volumetric changes, eliminating any contrast-induced effects (Reichenauer *et al.*, 2008 ▸). Moreover, SANS can be directly combined with *in situ* dilatometry, measuring the macroscopic length change of the sample simultaneously, with the two techniques probing essentially the same sample volume.

Here, we present experimental data from applying a new *in situ* setup for combined SANS and dilatometry to determine adsorption-induced deformation at two length scales simultaneously. We employ water vapour adsorption into a silica monolith with hierarchical porosity (disordered macropores and hexagonally ordered cylindrical mesopores). For each selected water vapour pressure, the macroscopic length change of the sample and the radial strain of the ordered cylindrical mesopore lattice are determined by dilatometry and SANS, respectively. In addition, we demonstrate that the incoherent scattering from water can easily be used to determine an adsorption/desorption isotherm of the sample under investigation.

## 2. Experimental   

**Figure 1:**
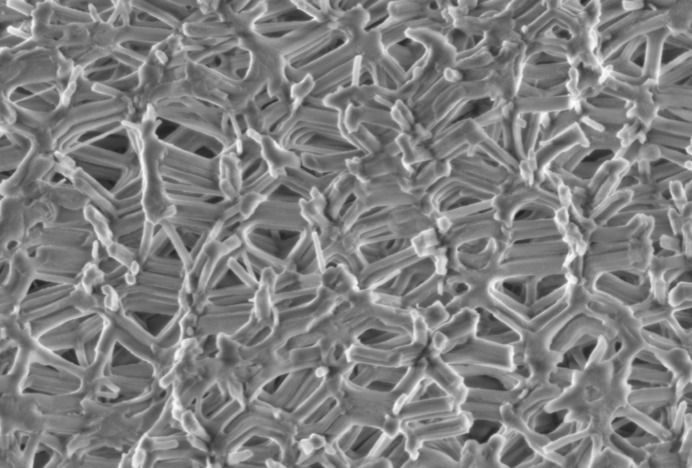
Scanning electron microscopy image of the hierarchical porous silica sample, showing the framework built by struts which contain hexagonally ordered mesopores. The empty space in between the struts in the size regime of 100 nm–1 µm is attributed to macropores.

The sample analysed was a monolithic silica with hierarchical porosity, consisting of an open network of macropores (according to IUPAC, radius *r* > 50 nm) and struts with hexagonally ordered cylindrical mesopores (2 < *r* < 50 nm), as shown in Fig. 1 ▸. The synthesis protocol was first introduced by Brandhuber *et al.* (2005 ▸) and is described only very briefly here. Wet gels were prepared by mixing tetra­kis­(2-hydroxyethyl)orthosilicate with an aqueous solution of Pluronic P123 in 1 *M* HCl in a weight ratio of Si/P123/HCl = 8.4/30/70. The homogenized sol was transferred into plastic moulds and aged at 313 K for 2 h. After extruding the gel into the final cylindrical shape with a diameter of 5 mm, it was subjected to further ageing at 313 K for 7 d. The resulting wet gels were demoulded, washed in ethanol (five times within 3 d) and dried with supercritical CO_2_ (*T*_c_ = 304.18 K; *P*_c_ = 7.38 MPa). This procedure resulted in a monolithic silica sample with a density ρ = 0.42 g cm^−3^, a surface area of 211 m^2^ g^−1^ and a mesopore diameter of 5.3 nm. The latter two values were determined from the nitrogen adsorption isotherm (77 K) *via* BET (Brunauer–Emmett–Teller) analysis (Brunauer *et al.*, 1938 ▸) for the determination of the specific surface area and the Gurvich rule (Gurvich, 1915 ▸) for the respective specific pore volume required for the calculation of the pore size assuming cylindrical pore geometry. No micropores (<2 nm) were detected in the sample, but some organic residues can be expected at the mesopore surface and within the mesopore walls owing to the absence of a calcination step.

The water vapour isotherm relevant for the actual *in situ* experiment had been determined in advance in a commercial gravimetric laboratory adsorption system (SPS11-10µ, ProUmid).

The adsorptive for the *in situ* experiment was a mixture of 91.95 wt% water (H_2_O) prepared according to ISO 3696:1987 (grade 1) and 8.05 wt% heavy water (D_2_O) (AcroSeal, Acros Organics with a purity of 99.95%), leading to a net zero coherent scattering length density of the adsorbate (zero-SLD).

A rectangular (5 mm height and 3 mm width) shaped sample of 1.4 mm thickness was first cut from the cylindrical monolith with a diamond saw, parallel to the cylinder axis. Subsequently, the silica sample was aged in water for 3 weeks at 323 K and eventually conditioned for another 2 weeks in zero-SLD water. The latter step was applied to ensure that all exchangeable hydrogen-containing groups within the silica were adjusted to the same hydrogen/deuterium ratio as in the adsorptive.

**Figure 2:**
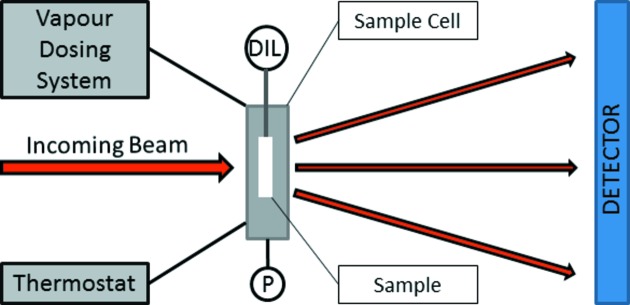
*In situ* adsorption setup combining a vapour dosing system (gas manifold) with the sample cell. P represents the pressure gauge connected directly to the sample cell. DIL stands for the vertically aligned dilatometer connected *via* a pushrod to the top of an essentially free-standing rectangular sample. The temperature of the water-cooled sample holder is kept at 290.2 ± 0.1 K with a thermostat.

SANS experiments were performed at the SANS-1 instrument at the Heinz Maier-Leibnitz Zentrum (MLZ) in Munich, Germany (Heinz Maier-Leibnitz Zentrum *et al.*, 2015 ▸; Mühlbauer *et al.*, 2016 ▸). A custom-built *in situ* sample cell was connected to an in-house-designed water vapour dosing system operating with water vapour rather than using a carrier gas (see scheme in Fig. 2 ▸). The cell is designed for combined *in situ* SANS and dilatometry on thin monolithic samples. The relative vapour pressure in the thermostated cell (±0.1 K) can be measured with an accuracy of ±0.15%. The sample is free to expand and the construction of the cell ensures the sample is positioned in a defined way with respect to the pushrod of the vertically aligned dilatometer (see Fig. 2 ▸). The sample holder was designed for samples with a thickness greater than 500 µm and 7–9 mm in diameter and was adapted especially for the used sample.

**Figure 3:**
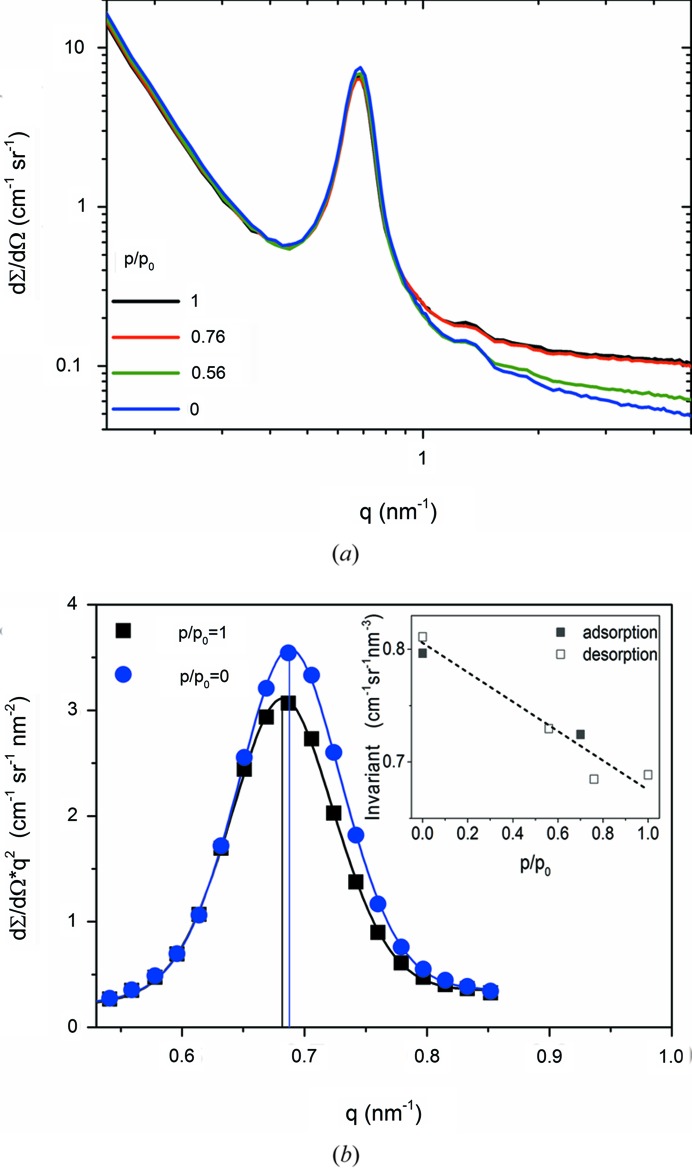
(*a*) *In situ* SANS differential scattering cross section taken during water vapour desorption for different relative vapour pressures, where the value of one corresponds to the bulk water vapour pressure of 19.4 mbar (1940 Pa) at the given temperature of 290.2 K. (*b*) First-order Bragg peak after subtraction of the incoherent scattering and the cross section multiplied with *q* ^2^ (Kratky plot) for the empty and the completely filled sample. The full lines are fits using a pseudo-Voigt function. A peak shift is indicated by the vertical lines at the respective peak maximum. The inset in Fig. 3 ▸(*b*) shows that the integrated cross section over the whole reciprocal space (scattering invariant) changes roughly linearly with relative pressure.

Prior to the *in situ* experiment, the sample was moved from a closed vessel with a zero-SLD water reservoir into a small laboratory vacuum oven and degassed under vacuum [<10^−3^ mbar (0.1 Pa)] at 373 K for 3 h. Eventually, the oven was vented with dry N_2_ gas and the still hot sample was quickly transferred into the *in situ* cell. After evacuation of the cell the sample was heated to 323 K for another hour and afterwards slowly cooled to the temperature of the experiment (290 K). *In situ* SANS patterns (see Fig. 3 ▸ *a*) were collected for different water vapour pressures: three during adsorption and four during desorption. Sample equilibration times after each vapour dosing step and before the start of SANS data collection were typically 30–60 min. The sample height was recorded by the dilatometer and averaged for each individual pressure step over the entire SANS measurement. Data collection times for SANS were 20 min each for the two configurations (1.1 and 5 m sample–detector distance at a neutron wavelength of 0.55 nm). The sample transmission was also determined for each pressure step by measuring the 1000-fold attenuated direct beam with and without the sample in the beam path for 2 min each. Spatial sensitivity inhomogeneities of the detector were corrected and the scattered intensity converted to the differential scattering cross section in absolute units using the isotropic scattering of a 1 mm-thick H_2_O sample. Subsequently, the data were radially averaged, and the two instrument configurations were merged together to obtain scattering cross sections 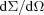 as a function of the scattering vector length *q* between 0.1 and 5 nm^−1^, where 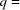 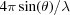, 2θ being the scattering angle and λ the neutron wavelength.

## 3. Results   

**Figure 4:**
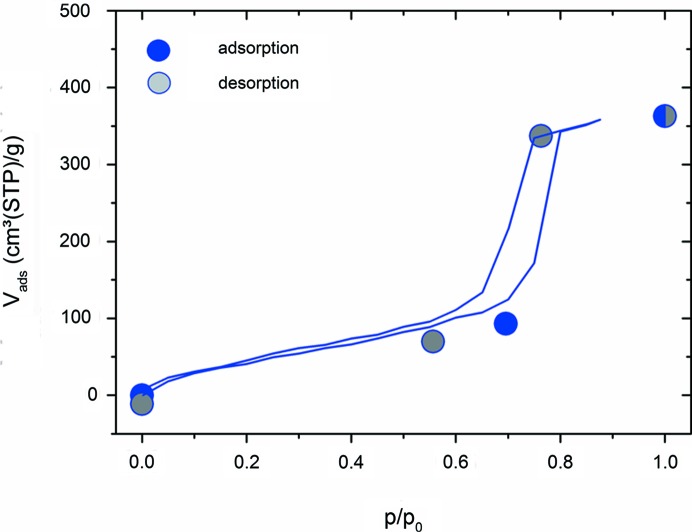
Water vapour isotherm of the sample measured *ex situ* with a gravimetric adsorption instrument (full lines). The symbols represent the adsorbed specific volume of water from the incoherent scattering of zero-SLD water directly obtained from the SANS data as described in the text. The error bar estimated from the counting statistics is smaller than the size of the symbols.

SANS differential scattering cross sections are shown in Fig. 3 ▸(*a*) for different relative pressures along the desorption branch of the water vapour isotherm (see Fig. 4 ▸). The data show a sharp Bragg peak at a scattering vector length 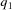 = 0.68 nm^−1^ and two further smaller peaks at 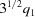 and 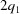, corresponding to diffraction from the two-dimensional hexagonal mesopore lattice with a lattice parameter *a* = 10.7 nm. For *q* values below 1 nm^−1^, the intensity is very similar for the different relative pressures, as would be expected from the coherent scattering of a porous sample with different amounts of zero-SLD water. It is noted, however, that the peak height changes slightly with pressure. The scattering signal is superimposed by an incoherent background increasing with the amount of zero-SLD water adsorbed.

For *q* > 1 nm^−1^, the strong incoherent scattering from H_2_O clearly indicates the different degrees of water filling. The constant part of the scattering intensity at large *q* can be determined by using Porod’s law superimposed by a constant background: 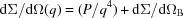. In principle, the incoherent scattering contribution expected from the water in the pores can be calculated explicitly from the adsorption isotherm in Fig. 4 ▸, which was measured *ex situ* prior to the experiment. We choose the opposite way and use the incoherent scattering to reproduce the adsorption isotherm as depicted in Fig. 4 ▸. For this, the scattering of a *t*_W_ = 1 mm-thick sample with pure zero-SLD water was measured in a quartz cuvette and background corrected to obtain the incoherent scattering of zero-SLD water, 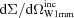. The work of Grillo (2008 ▸) shows that the multiple scattering of water has negligible influence on the SANS data at high *q* values and is therefore not regarded here. The incoherent scattering contribution of water to the sample scattering as a function of relative water pressure is given by 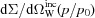 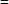 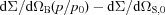, with 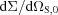 the sample-related scattering background at *p*/*p*_0_ = 0. From this, for each water vapour pressure step the mass of the adsorbed water can be calculated. This is done by multiplying the incoherent scattering cross section of the sample normalized to the incoherent cross section of a 1 mm-thick layer of zero-SLD water with the volume of the sample in the beam (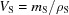) and the density of the water ρ_W_ (1.01 g cm^−3^). In derivation of equation (1) we have used the fact that the thickness of water in the sample per total sample thickness is equal to the volume of the water per total sample volume, since the area of the neutron beam is kept constant. With this, the adsorption isotherm, *i.e.* the adsorbed water volume per unit mass of sample 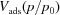, is obtained using the molar mass (*M*_W_ = 18.18 g mol^−1^) of zero-SLD water, the sample density (ρ_S_ = 0.421 g cm^−3^), and the volume of an ideal gas at standard temperature and pressure (STP, *V*_mol_ = 22414 cm^3^ mol^−1^):
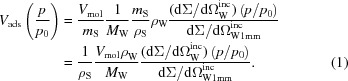



Fig. 4 ▸ demonstrates that the incoherent scattering can be used to reproduce the (*ex situ*) adsorption isotherm very well. Small deviations may occur, due to equilibration issues and due to systematic errors in the sample thickness determination. This means that for water adsorption the incoherent neutron scattering provides an elegant approach for determining adsorption isotherms, if the coherent and the incoherent scattering contributions can be separated reliably.

In the following the coherent scattering as a function of relative pressure is analysed in detail. Fig. 3 ▸(*b*) shows two effects of water adsorption on the first Bragg peak. First, the peak shifts as expected owing to water-vapour-adsorption-induced deformation (Balzer *et al.*, 2015 ▸; Prass *et al.*, 2009 ▸). Secondly, there is also a noticeable change of the peak intensity after subtraction of the incoherent scattering. This is at first unexpected since the adsorption and condensation of zero-SLD water within the mesopores should leave the intensity essentially unchanged. An attempt to explain this effect will be given in §4.

The deformation of the mesopore lattice due to water vapour adsorption and desorption was calculated by determining the relative shift of the peak position with respect to its position at *p* = 0 (Günther *et al.*, 2008 ▸),
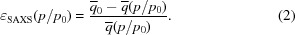
Here the peak position 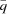 was determined from the fit of a pseudo-Voigt function to the first Bragg peak in a Kratky plot (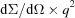 *versus q*) in the *q* region shown in Fig. 3 ▸(*b*), including a power-law contribution from the diffuse coherent scattering:
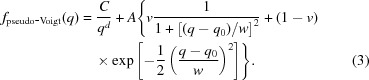
The Lorentz/Gauss ratio *v* in the fitting function *f*_pseudo-Voigt_ was determined for one curve and then kept constant for all other curves; all other parameters (position 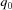, width *w* and peak height *A*, as well as the two parameters *C* and *d*) were free fitting parameters.

**Figure 5:**
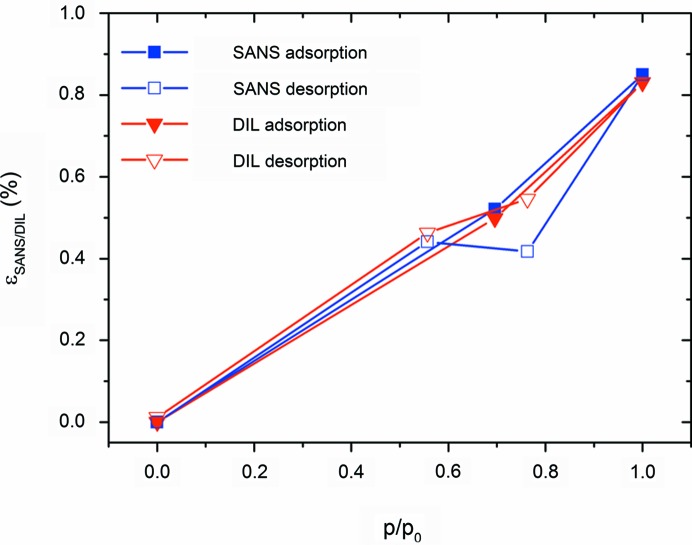
Strain isotherms from dilatometry (red) and from SANS (blue).

The corresponding macroscopic sample deformation was obtained from the relative height change of the sample sheet measured by dilatometry:
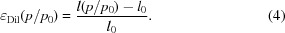
The strain isotherms (*i.e.* the strain as a function of relative water vapour pressure) from SANS and dilatometry are depicted in Fig. 5 ▸. The maximum strains for the completely filled sample are about 0.85%. They agree almost perfectly for the two independent methods, and so do the data along the adsorption branch. The only obvious deviation between the two data sets is observed in the region of capillary evaporation along the desorption branch, where the strain from SANS shows a clear dip. Hence, despite the low number of measurement points the strain isotherm obtained from the SANS data shows a hysteresis similar to the adsorption isotherm (see Fig. 4 ▸), while the strain from dilatometry does not.

## 4. Discussion   

In our previous work (Balzer *et al.*, 2015 ▸), we have for the first time combined SAXS and dilatometry data to determine adsorption-induced deformation on a silica sample with hierarchical porosity. Those measurements were performed separately on different samples from the same batch using *n*-pentane as an adsorbate. Here we report a combined investigation of adsorption-induced deformation by simultaneous *in situ* SANS and dilatometry using a custom-built setup specifically designed for this purpose. There are several advantages of this new approach: (i) The same sample is measured simultaneously by both techniques, circumventing any artefacts related to sample ageing, pressure and/or temperature differences *etc*. (ii) Since an almost identical sample volume is probed by the two techniques, possible sample inhomogeneities do not blur the results from the two techniques. (iii) The use of zero-SLD water avoids any apparent strains due to the change of the scattering contrast which is of particular importance in the region of capillary condensation (Prass *et al.*, 2012 ▸).

Previously (Balzer *et al.*, 2015 ▸), we observed a much stronger ‘dip’ in the SAXS desorption strain isotherm at capillary evaporation as compared with dilatometry, which is quite consistent with this work. This dip is related to the negative Laplace pressure at capillary evaporation arising from a curved gas–liquid interface, leading to sample contraction (Prass *et al.*, 2009 ▸). This effect is superimposed by the so-called disjoining pressure change at the solid–liquid interface, which leads generally to sample expansion with increasing relative pressure due to the decrease of the surface energy upon adsorption. The general trend of the adsorption strain to increase with vapour pressure, interrupted by a sudden decrease at capillary condensation and subsequent further increase, is in good agreement with many investigations (Gor *et al.*, 2015 ▸; Grosman *et al.*, 2015 ▸; Prass *et al.*, 2012 ▸; Dolino *et al.*, 1996 ▸; Lakhanpal & Flood, 1957 ▸; Amberg & McIntosh, 1952 ▸). The difference between the dilatometry and SANS results can be rationalized on the basis of the following considerations, taking the hierarchical sample structure (see Fig. 1 ▸) into account. While SANS probes the radial expansion of the struts, dilatometry is sensitive to the macroscopic deformation, depending on both the radial and the axial strains in a non-trivial way. In a recent paper, Balzer *et al.* (2017 ▸) have shown that the axial and radial stresses upon N_2_ adsorption in a single cylindrical pore may differ considerably. By estimating strains using simplified mechanical models and finite element calculations it was demonstrated that radial strains should indeed show a much more pronounced dip at capillary condensation than axial strains, while both should reach the same strain level at complete mesopore filling. Assuming the dilatometry data to be dominated by the axial strains – which is reasonable since the aspect ratio of strut length to strut diameter is about 4 – this is qualitatively what we see in Fig. 5 ▸. Note, however, that the maximum strain reported by Balzer *et al.* (2017 ▸) is smaller by roughly a factor 50 than the one in the present work. This huge difference cannot be explained solely by the different adsorptive, nor does it seem likely that the wall stiffness in the present sample should be so much lower. We rather expect that the huge strain observed experimentally is related to the organic filled micropores present in the system. The large impact of micropore-induced adsorption strain has been demonstrated experimentally for microporous carbons (Balzer *et al.*, 2011 ▸). Therefore, we refrain from making any quantitative statements based on theory at this point, since the adsorption of water vapour in a non-calcined sample with residual organics is far from the model case presented by Balzer *et al.* (2017 ▸), *i.e.* N_2_ adsorption in a purely mesoporous sample.

Finally, we discuss the change of the SANS intensity upon water vapour adsorption. From the completely empty to the completely filled state the integrated intensity decreases by more than 12% [see the inset in Fig. 3 ▸(*b*)]. This is much more than would be expected from the change of pore volume due to adsorption-induced deformation, since the maximum radial strain in Fig. 5 ▸ translates to a maximum area change of the strut cross section of 2 × 1.7%. The sample was conditioned in zero-SLD water before the *in situ* adsorption experiments. Therefore, hydrogen in ≡Si—OH silanol surface groups or in the organic residues should have been replaced by the amount of deuterium corresponding to zero-SLD, and therefore should not lead to a contrast change unless the change in vapour pressure initiates an increasing uptake of water molecules in the pore wall. We believe the surface of the pores is covered with ethoxy groups from the washing steps before drying as well as with residual surfactant moieties. Calcination at 773 K shows a mass loss of roughly 30 wt%, proving the high mass and volumetric contribution of the organics. Incorporation (*i.e.* absorption) of water molecules in the organic phases will have a twofold effect. First, it will result in swelling of the organic phase towards the pore space, thus reducing the effective porosity, and, second, it will reduce the contrast between the pore wall and the pore. In addition, isotope effects have been reported for the absorption of water into polymers, which might partly explain the changes in the invariant observed in Fig. 3 ▸(*b*) (Delajon *et al.*, 2009 ▸). The assumption of swelling is supported by the fact that the integrated differential cross section changes monotonically and smoothly with relative pressures. However, since we have no reliable information on the density and composition of the organic residues, no quantitative estimate can be made at this point.

## 5. Conclusion   

Water-adsorption-induced deformation of a silica monolith with hierarchical porosity was determined simultaneously at the macroscopic and the nanometre scale by combining *in situ* dilatometry and SANS using a custom-made sample environment. This approach allows extraction of highly accurate complementary data sets that can be reliably compared as they are taken on essentially the same sample volume. Moreover, the use of zero-SLD water avoids any artefacts due to contrast-induced shifts of the pore lattice reflections, which are known to obscure the results from SAXS measurements. It is demonstrated that the water adsorption isotherm of the sample can be reliably extracted from the incoherent scattering from H_2_O. The determined adsorption-induced strains reveal similar deformation on the macroscopic and the nanometre scale in the regime of surface adsorption. In contrast, in the regime of capillary evaporation the hierarchical silica shows clearly different deformation effects at the two length scales. This is attributed to differences in the physical origin of the strains measured by SANS and dilatometry, being sensitive to strain in different directions within the struts. These results are qualitatively in line with theoretical predictions for adsorption-induced deformation of such a type of hierarchical porous materials.
